# Analytical Platform for the Study of Metabolic Pathway of Propyl Propane Thiosulfonate (PTSO) from *Allium* spp.

**DOI:** 10.3390/foods12040823

**Published:** 2023-02-15

**Authors:** María García-Nicolás, Marta Pastor-Belda, Natalia Campillo, María Jesús Rodríguez-Sojo, Antonio Jesús Ruiz-Malagón, Laura Hidalgo-García, Paloma Abad, José Manuel de la Torre, Enrique Guillamón, Alberto Baños, Julio Gálvez, Pilar Viñas, Natalia Arroyo-Manzanares

**Affiliations:** 1 Department of Analytical Chemistry, Faculty of Chemistry, Regional Campus of International Excellence “Campus Mare Nostrum”, University of Murcia, E-30100 Murcia, Spain; 2 Department of Pharmacology, Instituto de Investigación Biosanitaria (ibs. GRANADA), Center for Biomedical Research (CIBM), University of Granada, E-18071 Granada, Spain; 3 Department of Inorganic and Organic Chemistry, Campus of Lagunillas, Universidad de Jaén, E-23071 Jaén, Spain; 4 Department of Microbiology, University of Granada, Fuente Nueva s/n, E-19071 Granada, Spain

**Keywords:** propyl propane thiosulfonate, rats, metabolisation, in vivo study, analytical platform

## Abstract

The present work is focused on the development of an analytical platform to elucidate the metabolic pathway of PTSO from onion, an organosulfur compound well-known for its functional and technological properties and its potential application in animal and human nutrition. This analytical platform consisted of the use of gas chromatography–mass spectrometry (GC-MS) and ultra-high performance liquid chromatography quadrupole with time-of-flight MS (UHPLC-Q-TOF-MS) in order to monitor volatile and non-volatile compounds derived from the PTSO. For the extraction of the compounds of interest, two different sample treatments were developed: liquid–liquid extraction (LLE) and salting-out assisted liquid–liquid extraction (SALLE) for GC–MS and UHPLC-Q-TOF-MS analysis, respectively. Once the analytical platform was optimised and validated, an in vivo study was planned to elucidate PTSO metabolisation, revealing the presence of dipropyl disulfide (DPDS) in liver samples with concentrations between 0.11 and 0.61 µg g^−1^. The DPDS maximum concentration in the liver was observed at 0.5 h after the intake. DPDS was also present in all plasma samples with concentrations between 2.1 and 2.4 µg mL^−1^. In regard to PTSO, it was only found in plasma at times above 5 h (0.18 µg mL^−1^). Both PTSO and DPDS were excreted via urine 24 h after ingestion.

## 1. Introduction

*Allium* essential oils contain a great number of organosulfur compounds (OSCs), which are related to their well-known antimicrobial, antiviral, antiprotozoal, antifungal, and antioxidant properties [1]. Among them, propyl propane thiosulfonate (PTSO) is one of the compounds responsible for the odour of freshly cut onion and is also the most studied thiosulfonate present in onions. It has been used in different applications due to its multiple health benefits [2], including modulation of inflammatory response and improvement of gut health. In addition, PTSO has also shown interesting properties with potential applications in human nutrition, such as its ability to attenuate metabolic alterations in obese mice, reduce weight gain, and improve plasma markers related to glucose and lipid metabolisms [3,4,5,6]. Moreover, its antioxidant and antimicrobial activities against Enterobacteriaceae, Escherichia coli, Salmonella enterica, Campylobacter jejuni [5,7], *Eimeria acervulina*, and *Clostridium* spp. have been reported [4]. Thus, PTSO is capable of inhibiting the growth of Gram-negative and Gram-positive bacteria and also moulds and yeasts in animal models [8]. In addition, an in vitro study has recently demonstrated its antibacterial activity when compared with antibiotics commonly used against bacterial contamination in human clinical samples [9]. Moreover, in vitro anti-candidiasis activity through aerial diffusion of PTSO has been proved [10].

Following the European Union’s ban on the use of antibiotic growth promoters in animal production, there is a clear demand for natural additives capable of guaranteeing performance in animal production while avoiding the adverse effects of antibiotics [5]. In this sense, the use of PTSO as a supplement to animal feed has demonstrated some benefits that could be related to the inhibition of methane production during rumen fermentation [11] and its anti-inflammatory properties [3], also guaranteeing the safety and sensory attributes of animal products. Moreover, the addition of *Allium* extract rich in PTSO at 5 mg kg^−1^ improves pig microbiota, and it would be a good supplement to increase the pig growth rate [9]. In addition, the intake of PTSO in the diet has been shown to modulate the intestinal microbiota of piglets and laying hens, improving their performance [12]. PTSO toxicity was also evaluated in humans using cytotoxicity assays [13], in vitro and in vivo genotoxicity, and in vitro mutagenicity assays [14,15]. The acute toxicity of PTSO was also evaluated in vivo [13], proposing the maximum tolerable dose (MTD) at 55 mg kg^−1^. An in vivo study in rats, administering PTSO for 90 consecutive days, confirmed the safety profile of PTSO for food applications since the dose corresponding to the no-observed-adverse-effect level (NOAEL) was equal or greater than MTD (55 mg kg^−1^ day^−1^).

In order to ensure the safety of the use of PTSO as a food or feed ingredient, the assessment of safety for consumers is essential. For this purpose, it is necessary to consider the potential toxicity of the additive [8,13], consumer exposure to higher concentration levels, and the metabolic fate and residues of the additive in laboratory animals.

For the metabolomic study, it is necessary to carry out in vivo studies and monitor PTSO and derivative compounds in biological fluids and tissues. Nevertheless, no in vivo studies for PTSO metabolisation have been found in the literature to date. However, in vitro and in vivo metabolic studies have been developed by other authors to elucidate the metabolic pathway of dipropyl disulfide (DPDS) [16,17], which always appears in very low concentrations besides PTSO [2]. Teyssier et al. [16] used Wistar rats housed individually in wire cages with light–dark cycles and fed for 4 weeks. After this, intragastric administration of DPDS was applied to each rat during the established period of time before their sacrifice. Rat liver cell subfractions and isolated perfused rat livers were analysed using gas chromatography with mass spectrometry (GC–MS), founding several following compounds: dipropyl thiosulfinate (DPDSO); propyl mercaptan (PM); and propyl glutathione sulphide (PGS), which were detected in the presence of liver subcellular fractions, whereas PM, PGS, methyl propyl sulphide (MPS), and methyl propyl sulfone (MPSO_2_) were formed in the perfused liver.

Furthermore, Germain et al. [17] fed Wistar rats for 1 week before starting the experiment. A single dose of DPDS was administered by gastric intubation at 200 mg kg^−1^ body weight, and some rats were euthanized at different times up to 48 h after oral dosing. PM, MPS, MPSO, and MPSO_2_ were identified using GC-MS as DPDS metabolites in the stomach, intestine, liver, and plasma.

Furthermore, it has been demonstrated that PTSO reacts with cysteine and glutathione, both present in a wide range of biological tissues, resulting in s-propyl mercaptocysteine (CSSP) and s-propyl mercaptoglutathione (GSSP) as products. The development of analytical methods that allow the determination of PTSO and its metabolites at the low expected concentrations is essential. Due to its different physicochemical properties, the analysis implies the combination of different analytical techniques in a platform that allows to monitor as many PTSO metabolites as possible, including volatile and non-volatile compounds.

The analytical methods for the determination of PTSO and/or its derivatives, such as DPDS, PTS, CSSP, and GSSP, are mainly based on liquid chromatography (LC) coupled to different detectors as diode array (DAD) [15,18,19,20] or tandem MS using electrospray ionisation (ESI-MS/MS) [11,19,20,21,22], and GC–MS [2,16,17].

These methods have been applied for the analysis of animal feed and biological fluids, tissues, or food derived from animals that have been fed with PTSO, such as milk and eggs. For the treatment of these samples, solid–liquid extraction (SLE) or liquid–liquid extraction (LLE) step using different solvents, such as methanol (MeOH), acetonitrile (ACN), acetone, and dichloromethane, were commonly used after protein precipitation using trichloroacetic acid or formic acid (FA) when biological samples, such as blood, plasma, and tissues, are analysed [2,11,16,17,18,20,23]. In addition, other techniques, such as dispersive liquid–liquid microextraction (DLLME) [2], have also been used for the analysis of animal feed in order to clean the samples and pre-concentrate the analytes.

The aim of the present study is to develop and validate an analytical platform to obtain an approach to the metabolic pathway of PTSO, which has demonstrated several beneficial properties and high stability being a very interesting compound of great interest to be exploited in the food and feed industry. The analytical platform is based on the combination of LLE followed by GC–MS and salting-out assisted liquid–liquid extraction (SALLE) coupled by ultra-high performance liquid chromatography quadrupole with time-of-flight hybridisation (UHPLC-Q-TOF-MS). Finally, an in vivo study after oral administration of PTSO is proposed as being of utmost relevance and remaining unexplored so far.

## 2. Materials and Methods

### 2.1. Reagents and Standards

Standards with purities higher than 99% of DPDS, PTS, PTSO, CSSP, and GSSP were provided by the DMC Research Center (Granada, Spain). Standard solutions of each compound at 1000 mg L^−1^ were prepared in MeOH purchased from ChemLab (Zedelgem, Belgium) and stored at −20 °C. A working solution containing all compounds at 10 mg L^−1^ was daily prepared by diluting the previous solutions in MilliQ water (Millipore, Bedford, MA, USA). ACN provided by ChemLab, sodium chloride, FA, and ethyl acetate (EA) from Sigma Aldrich (Steinheim, Germany) were used to extract the compounds from the biological samples. Helium, provided by Air Liquide (Madrid, Spain), was used as a carrier gas in GC–MS instrumentation.

Rabbit liver purchased from a local butcher shop, human urine, and freeze-dried synthetic pig plasma supplied by Sigma Aldrich were used for method optimisation.

### 2.2. Instrumentation

A GC Agilent 6890 N equipped with a split/splitless injector coupled to a 5973 mass spectrometer (Agilent Technologies, Santa Clara, CA, USA) with an electron impact ionisation source and a quadrupole analyser was used as GC–MS instrumentation. UHPLC-Q-TOF-MS analyses were carried out using an Agilent 1290 Infinity II Series UHPLC instrument (Agilent Technologies) with a binary pump. The LC system was coupled to an Agilent 6550 Q-TOF Mass Spectrometer (Agilent Technologies) equipped with an ESI source (Agilent Jet Stream Dual electrospray, AJS-Dual ESI). MassHunter Workstation Data Acquisition software (Agilent Technologies, Rev.B.08.00) was used for data processing.

For a sample treatment, a vortex shaker LLG-uniTEXER (Constanti, Tarragona, Spain) and a refrigerated centrifuge MPW-150R (Warsaw, Poland) were used.

### 2.3. GC–MS Analysis

The GC–MS methodology applied is based on the previous work developed by Pastor-Belda et al. with slight modifications. A HP-5MS ultra inert capillary column (Agilent, 5% diphenyl-95% dimethylpolysiloxane), with dimensions of 30 m length, 0.25 mm internal diameter, and 0.25 μm film thickness, was used as stationary phase. The mobile phase (He) was established at 1 mL min^−1^ in a constant flow mode. The sample (2 μL) was injected at 280 °C in splitless mode. The oven programme, with a total time of 9.5 min, consisted of an isothermal stage at 50 °C for 1 min and two linear temperature gradients. The first one achieved 160 °C at a rate of 25 °C min^−1,^ and after that, the temperature increased up to 250 °C at a rate of 30 °C min^−1^, maintaining this temperature for 1.1 min. GC–MS transfer line was maintained at 300 °C. Mass spectrometer ionisation source operated at a voltage of 70 eV with a temperature of 230 °C, and the quadrupole temperature was fixed at 150 °C. A mass spectrometer was used in scan mode to be able to search and identify other compounds for which no standard was available. Table 1 shows the retention times, as well as the identification and quantification ions used for PTSO and DPDS for which standards were available.

### 2.4. UHPLC-HRMS Analysis

UHPLC separation conditions are based on the work developed by Abad et al. [20] using a ZORBAX RRHD Eclipse Plus C18 column (100 mm × 2.1 mm, 1.8 μm) from Agilent Technologies (Diegem, Belgium) with a 0.3 µm in-line filter. The mobile phase consisted of a 0.05% *v*/*v* formic acid solution (Solvent A) and MeOH (Solvent B). The elution gradient was as follows: 0–0.5 min: 10% B; 2–3.5 min: 100% B. Finally, the initial conditions were re-established in 0.5 min and maintained for 2 min to equilibrate the column. The mobile phase flow rate was set at 0.4 mL min^−1,^ and the column temperature was maintained at 35 °C. The injection (20 μL) was carried out using an autosampler, thermostated at 5 °C, using 2 mL vials provided with 250 µL capacity micro-inserts.

The Q-TOF system was operated in positive ESI mode polarity with the following operating parameters: capillary voltage, 4000 V; nebuliser gas pressure, 40 psi; drying gas flow, 16 L min^−1^; drying gas temperature, 150 °C; fragmentor voltage, 360 V; nozzle voltage, 500 V; 1 RF Vpp octapole, 750 V. Data acquisition was carried out in the 50–500 *m*/*z* mass range. Both untargeted and targeted acquisition modes were used. The untargeted acquisition combines Full MS and all-ion fragmentation mode at 10 and 40 V, where no precursor ion isolation is carried out. On the other hand, targeted acquisition (Targeted MS/MS) allows the isolation of the precursor and the fragmentation of the compounds at high resolution (40 V). Data were acquired in a 2 GHz extended dynamic range mode with 3 spectra, 333.3 ms/spectrum, and 2675 transients/spectrum.

The Q-TOF spectrometer calibration was performed using a dual ionisation source with an automatic calibrant supply system, which simultaneously introduced the flow from the chromatograph with small amounts (about 20 µL min^−1^) of a calibrant solution containing the reference mass 121.0509 Da in positive ESI mode.

Compound identification was carried out by comparison of retention times, exact mass, isotopic profile and mass spectra of available standards. For quantification purposes, the area obtained in the extracted ion chromatogram (EIC) for the protonated molecules, with a window of 0.01 Da, was used.

Table 1 shows the retention times, experimental and calculated *m*/*z* values, *m*/*z* error, and qualifier ions for three PTSO metabolites (PTS, CSSP and GSSP) for which the standards were available. The exact theoretical masses based on the formula were calculated using the molecular mass calculator tool included in MassHunter software. The instrumental error committed by the measurement of *m*/*z* was calculated as the difference between the experimental value and the theoretical value divided by the theoretical mass and multiplied by 10^6^.

### 2.5. In Vivo Study

The in vivo study has been carried out following the “Guide for the care and use of laboratory animals” of the National Institute of Health (USA), and the protocols have been approved by the Committee of Ethics of the University of Granada (Reference No. 28/03/2016/030). Adult female Sprague Dawley rats (240 g), provided by Charles River Laboratories (Les Oncins, France), were maintained individually in metabolic cages and in an air-conditioned atmosphere with a 12 h light–dark cycle and provided with food and tap water ad libitum. Rats were randomly assigned to different experimental groups: a control group (n = 10) and a treated group (n = 25), which received by oral gavage a single dose of PTSO (55 mg kg^−1^), suspended in sterile water. A single dose of 55 mg kg^−1^ was selected according to the previously described NOAEL value [8]. The control group was administered with the vehicle. Rats from the treated (n = 5) and control groups (n = 2) were anaesthetized with isoflurane; the blood was collected by cardiac puncture, and then the rats were sacrificed to obtain liver and faeces. This was performed at different times from the PTSO administration: (30 min, 2 h, 5 h, 24 h, and 48 h). For plasma collection, the blood was centrifuged at 3000× *g* for 10 min at 4 °C. All samples were stored at −20 °C until use. One day prior to the administration of PTSO and 24 and 48 h after, the urine was collected from animals from the different experimental groups.

### 2.6. Sample Treatment

**Figure 1:**
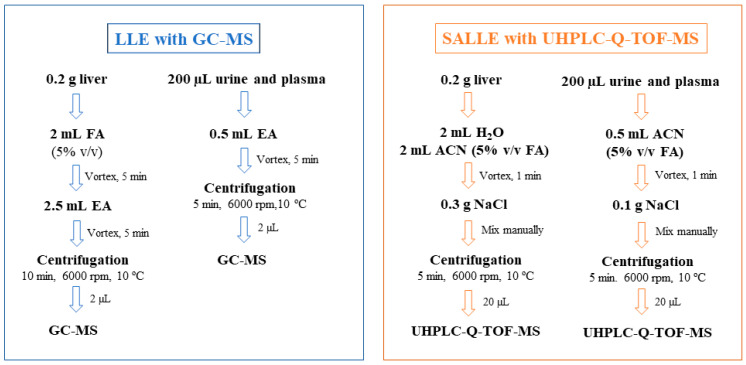
Scheme of sample procedures applied to the analytical platform.

Different sample treatments were carried out depending on the matrix and type of chromatography employed. LLE was used for GC–MS analysis, whereas SALLE with UHPLC-Q-TOF-MS analysis was used, as shown in Figure 1.

#### 2.6.1. LLE

Liver samples were manually crushed before analysis. Then, 0.2 g was weighed in a Falcon tube, and 2 mL of a 5% *v*/*v* FA aqueous solution was added and shaken by vortex for 5 min. Subsequently, 2.5 mL of EA was added and vortexed again for 5 min to promote the transfer of the analytes to the organic phase. A centrifugation step for 10 min at 6000 rpm and 10 °C was applied to separate organic and aqueous phases.

In the case of urine and plasma, 200 μL of the sample was placed into a 2 mL Eppendorf tube, and 0.5 mL of EA was added. The mixture was shaken by vortex agitation for 5 min and centrifuged for 5 min at 6000 rpm and 10 °C.

In all cases, after centrifugation, the supernatant (organic phase of EA) was collected, and 2 µL of filtered solution (0.22 µm nylon filters) was injected into the GC–MS system.

#### 2.6.2. SALLE

0.2 g of the crushed liver samples was placed in a Falcon tube. Then, 2 mL of water and 2 mL of ACN containing 5% *v*/*v* FA were added. After vortexing for 1 min, 0.3 g of NaCl was added and mixed manually, saturating the aqueous phase and resulting in a two-phase system (ACN and water saturated with NaCl) after centrifugation at 10 °C for 5 min at 6000 rpm.

For urine and plasma samples, a volume of 200 μL was placed into a 2 mL Eppendorf tube, and 0.5 mL of ACN containing 5% *v*/*v* FA was added. The mixture was vortexed for 1 min, and 0.1 g of NaCl was added and mixed manually. After that, the mixture was centrifugated for 5 min at 6000 rpm and 10 °C.

In all cases, the supernatant (organic phase of ACN) was collected, filtered (0.22 µm nylon filters), and 20 µL was injected into the UHPLC-Q-TOF-MS system.

## 3. Results and Discussion

### 3.1. Separation and Detection Conditions of PTSO and Its Metabolites

PTS and DPDS participate in the biosynthetic pathway for PTSO [10]. For that reason, they were included as possible derived metabolites in the PTSO metabolisation. In addition, it has been described that PTSO can react with other compounds, such as cysteine and glutathione, giving rise to the products CSSP and GSSP, respectively [20,22], so they were also included in this study.

The separation and detection conditions were established using PTSO and some of the previously mentioned derivatives (DPDS, PTS, CSSP, and GSSP), for which standard solutions were available. Due to the different physic-chemical characteristics of the compounds, their determination was possible thanks to the combination of two different analytical platforms, one for the analysis of PTSO and DPDS using GC–MS and another for the analysis of PTS, CSSP, and GSSP by UHPLC-Q-TOF-MS. Although both LC and GC–MS methods were reported to be appropriate for PTSO determination, in this case, the best results were obtained with GC–MS. This section may be divided into subheadings. It should provide a concise and precise description of the experimental results, their interpretation, as well as the experimental conclusions that can be drawn.

### 3.2. Sample Treatment Optimisation

Biological samples require an exhaustive extraction and clean-up treatment prior to chromatographic analysis. Sample treatment was optimised using PTSO and some of its possible metabolites (DPDS, PTS, CSSP, and GSSP), for which standard solutions were available. The matrices used for the method optimisations were rabbit liver, commercial lyophilized pig plasma (Sigma Aldrich P2891-10 mL, reconstituted in 10 mL of water) and human urine fortified at 500 ng g^−1^ or 500 ng mL^−1^, depending on the sample.

SALLE procedure was initially applied to the analysis of the samples in the biological tissue and fluids (plasma, urine and liver). This methodology, in addition to providing an optimal solvent for GC injection, allows the matrix clean-up, thus improving the selectivity of the method. The most important parameters affecting SALLE efficiency are aqueous phase volume, nature and volume of extractant solvent, and the amount of NaCl used, being, therefore, studied.

**Figure 2:**
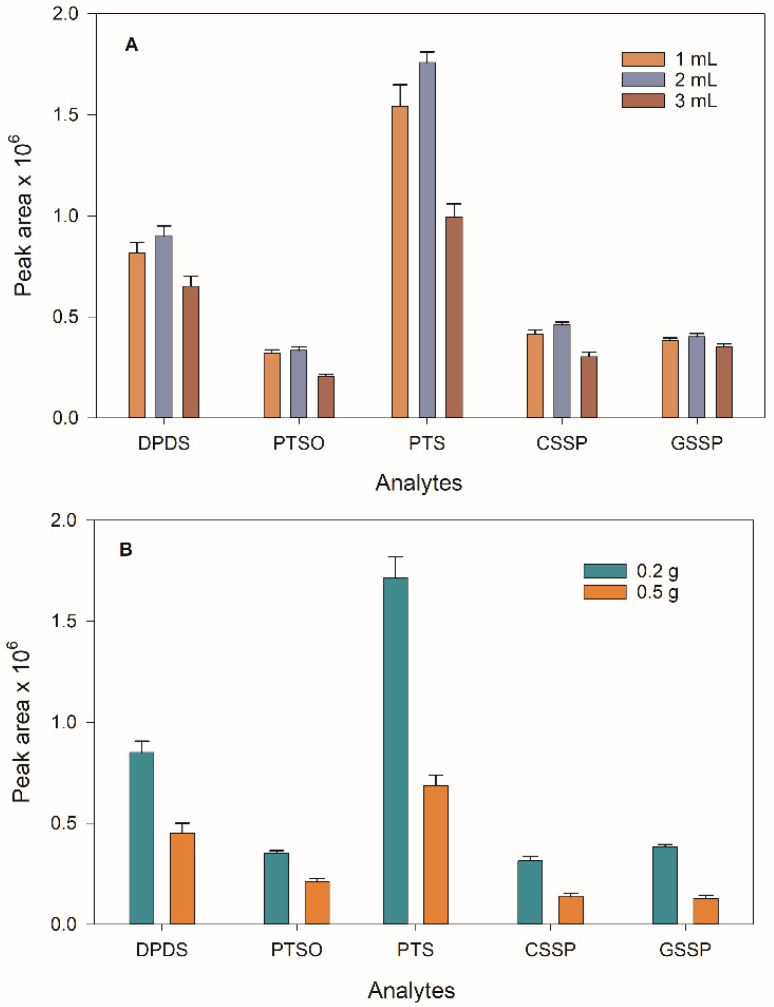
(**A**) Optimisation of ACN volume used for SALLE technique. (**B**) Signals obtained using different sample masses.

The optimisation of the extractant solvent for liver analysis was carried out by mixing 0.2 g of liver, 2 mL of water, the extractant solvent (ACN) and 0.3 g of NaCl. Three different volumes (1, 2 and 3 mL) were tested, and no significant differences were found between 1 and 2 mL. The use of 1 mL of ACN could provide higher preconcentration factors, but a greater matrix effect was observed, with analytical signals similar to those obtained with 2 mL. On the other hand, the use of 3 mL of ACN resulted in a signal decrease due to the dilution effect (Figure 2A). Therefore, a volume of 2 mL of ACN was finally chosen.

In the case of biological fluids, 2 mL urine or 200 µL plasma was mixed with ACN and 0.3 or 0.1 g NaCl, respectively. For urine analysis, the ACN volume was varied between 1–3 mL, again obtaining maximum signals for a 2 mL volume. Due to the lower volume of plasma used (200 µL), the volume of ACN was accordingly studied between 0.25 and 1 mL. Using 0.25 mL, no supernatant phase could be collected, whereas, for 0.5 and 1 mL, approximately 0.3 and 0.6 mL were collected, respectively. The increment of the supernatant phase volume resulted in a decrease in the signal, so the best extraction efficiency was obtained using 0.5 mL of ACN.

The extraction efficiency of the compounds by SALLE was also studied in acidic conditions. Therefore, the results obtained after the addition of ACN or ACN containing 5% *v*/*v* FA were compared, and an increase in the extraction efficiency using acid was observed for all biological matrices studied. Consequently, the solvent extract selected was ACN containing 5% *v*/*v* FA.

However, the recoveries of DPDS and PTSO from plasma samples using SALLE methodology and GC analysis were not completely satisfactory; thereupon, the LLE technique was tested as an alternative, using different extractant solvents, obtaining excellent recoveries for both compounds when using EA as extractant. The sensitivity of the method improved considerably compared to that obtained by the SALLE procedure, so the LLE technique was chosen for the GC–MS analysis.

The volume of EA was also optimised for the different matrices. For liver and urine, EA volume was studied in the 1–3 mL range. For liver samples, no supernatant phase was obtained with volumes lower than 2 mL, so finally, a volume of 2.5 mL was chosen for both matrices. The volume selected for the plasma samples was 0.5 mL, as already described for SALLE.

The presence of FA was also tested for the LLE technique. In this case, 2 mL of a 5% *v*/*v* FA aqueous solution was added to the liver sample; meanwhile, 10 and 100 µL of FA were directly added to plasma and urine samples, respectively. The results obtained with and without FA were compared, and it was proved that the extraction efficiency for the analytes was only improved for the liver samples. Thus, FA was only added for liver analysis.

Hence, two different sample treatment techniques, SALLE and LLE, were chosen for the determination of compounds by LC and GC, respectively.

**Figure 3:**
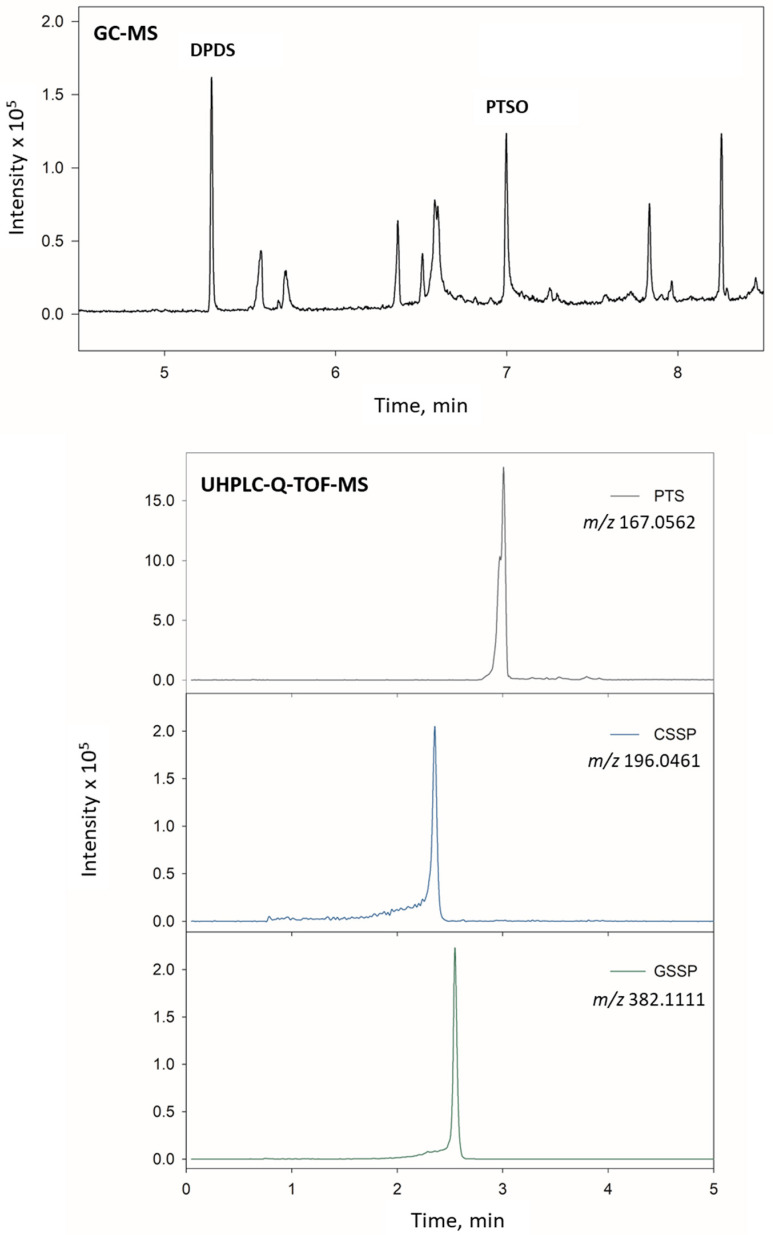
EICs obtained using GC–MS and UHPLC-Q-TOF-MS methodologies for a rat liver fortified at 500 ng g^−1^.

The sample amount was then optimised. For the liver analysis, two different masses (0.2 and 0.5 g) were studied, selecting 0.2 g due to a significant matrix effect found when using 0.5 g (Figure 2B). Urine volume was optimised up to 2 mL, and the results showed a continuous signal improvement. However, it was not possible to collect this amount from the rats, so a volume of 200 µL was finally selected. Similar behaviour was observed for the plasma samples, and a 200 µL volume was also selected. Figure 3 shows the extracted ion chromatograms (EICs) obtained for a liver sample fortified at 500 ng g^−1^ of all compounds and analysed using both methodologies. As can be seen, a good resolution was achieved for all the compounds in both UHPLC and GC techniques.

### 3.3. Validation of the Analytical Methods

The optimised methods were validated in terms of extraction efficiency, linearity range, limit of detection (LOD), limit of quantification (LOQ), and precision. All validation experiments were carried out using reference matrices: rabbit liver, commercial lyophilised pig plasma, and human urine.

#### 3.3.1. Validation of the LLE and GC–MS Procedure

Linearity was evaluated by obtaining calibration curves in the presence of a matrix at six different concentration levels (between 50–5000 ng g^−1^ for the liver and 50–5000 ng mL^−1^ for plasma and urine). The least square regression analysis showed satisfactory results, obtaining regression coefficients higher than 0.98 in all cases. Calibration curves in the absence of a matrix were also obtained using six different concentration levels between 5–500 ng mL^−1^ since, in the absence of a matrix, greater sensitivity was achieved. Significant differences were found between the slopes obtained in the absence and presence of matrix calibration curves (*p*-value < 0.001 using the one-factor ANOVA statistical test). Significant differences were also found between the different types of liver, urine, and plasma samples (*p*-value < 0.001). Nevertheless, when the slopes for each sample type were compared, the ANOVA test showed no significant differences. Therefore, matrix-matched calibration was proposed. Table 2 summarises the results obtained, including quantification slopes (average of n = 3 calibration lines of each type of sample) and linearity range.

The sensitivity of the method was assessed through the LOD and LOQ values, being obtained by applying the signal-to-noise ratio (S/N) criteria equal to 3 and 10, respectively (Table 2). The LOQ values for DPDS and PTSO were 43 and 125 ng g^−1^ for the liver, 20 and 100 ng mL^−1^ for urine, and 36 and 190 ng mL^−1^ for plasma, respectively, obtaining higher sensitivity for DPDS than for PTSO in all types of samples.

Relative standard deviation (RSD) was calculated to evaluate the precision of the method by means of repeatability studies (analyses performed on the same day) using liver, plasma, and urine samples. For this purpose, three aliquots (experimental replicates) of each type of sample fortified at two concentration levels, 500 and 3000 ng g^−1^ in liver samples and 500 and 3000 ng mL^−1^ in urine and plasma samples, were submitted to the GC–MS method. Each sample was injected in triplicate (instrumental replicates). The obtained results were in accordance with current legislation [24], with RSD values below 11% (Table 2).

#### 3.3.2. Validation of the SALLE and UHPLC-Q-TOF-MS Procedure

Calibration curves were performed in the absence and presence of matrix at six concentration levels, between 100–2000 ng g^−1^ for the liver and 25–1000 ng mL^−1^ for plasma and urine, to establish the linearity of the analytical method. Regression coefficients were higher than 0.98 in all cases (Table 3).

The comparison of the different slopes led to proposing a matrix-matched calibration for quantification purposes.

LOQ for PTS and CSSP in the liver analysis were 70 and 240 ng g^−1^, respectively. For urine and plasma, the most sensitive compound was also PTS, with LOQs of 20 and 33 ng mL^−1^ for urine and plasma, respectively. In addition, LOQ for GSSP were 140, 41, and 30 ng g^−1^ for liver, urine, and plasma, respectively (Table 3).

Repeatability studies were carried out using three aliquots of each matrix fortified at two concentration levels (500 and 1000 ng g^−1^ in the liver and 300 and 800 ng mL^−1^ in plasma and urine), each sample injected by triplicate. The RSD values were lower than 12% in all cases (Table 3), in accordance with current legislation [24].

### 3.4. In Vivo Animal Model for Establishing the Metabolic Pathway of PTSO

**Figure 4:**
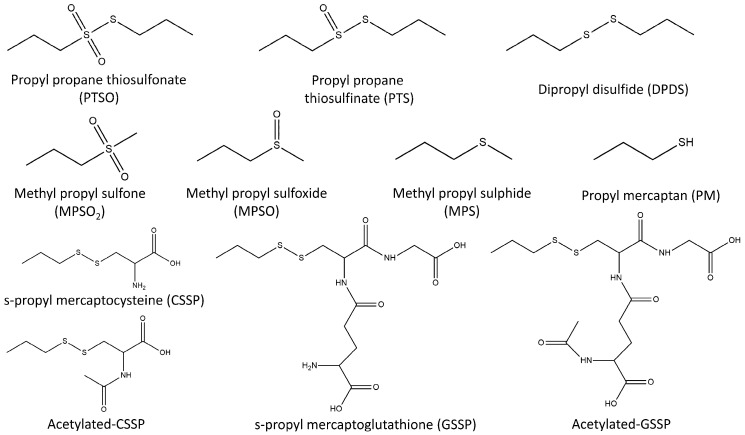
Structural formulas of the studied compounds and possible metabolic products.

The developed analytical platform was applied to verify the presence of the proposed organosulfur derivatives during the metabolisation of PTSO. For this purpose, samples obtained from in vivo study (30 liver, 30 plasma and 15 urine samples) were analysed by duplicate using both methodologies (GC–MS and UHPLC-Q-TOF-MS). In addition to PTSO and derivatives for which standards were available, other related compounds described in the literature were monitored. Specifically, PM, MPS, MPSO, MPSO_2_, acetylated-CSSP, and acetylated-GSSP were investigated (Figure 4) [16,17]. All of them were monitored using UHPLC-Q-TOF-MS (Table 4), and only the volatile compounds PM, MPS, MPSO, and MPSO_2_ were explored by GC–MS.

The results obtained for the group of rats fed with PTSO are shown in Table 5. Specifically, the mean concentration of the analytes at each time (n = 10, 5 rats at each time in duplicate) found in each matrix (liver, plasma, and urine) are shown.

Only DPDS was detected in liver samples at concentrations levels between 110–610 ng g^−1,^ with the highest concentration at 0.5 h, whereas both PTSO and DPDS were detected in urine and plasma samples. In plasma, DPDS was found at concentrations between 2100–2400 ng mL^−1^ from 0.5 h, and PTSO was present from 5 h at a practically constant concentration of 180 ng mL^−1^. Therefore, it would be convenient to propose a longer in vivo study.

**Figure 5:**
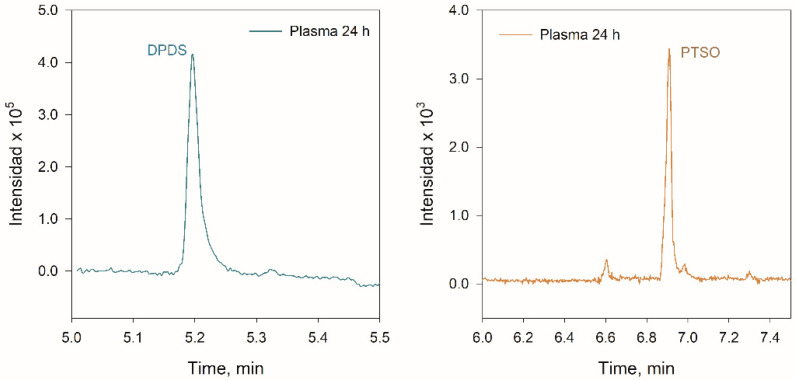
EICs obtained for DPDS and PTSO for a rat plasma sample obtained 24 h after PTSO ingestion.

**Figure 6:**
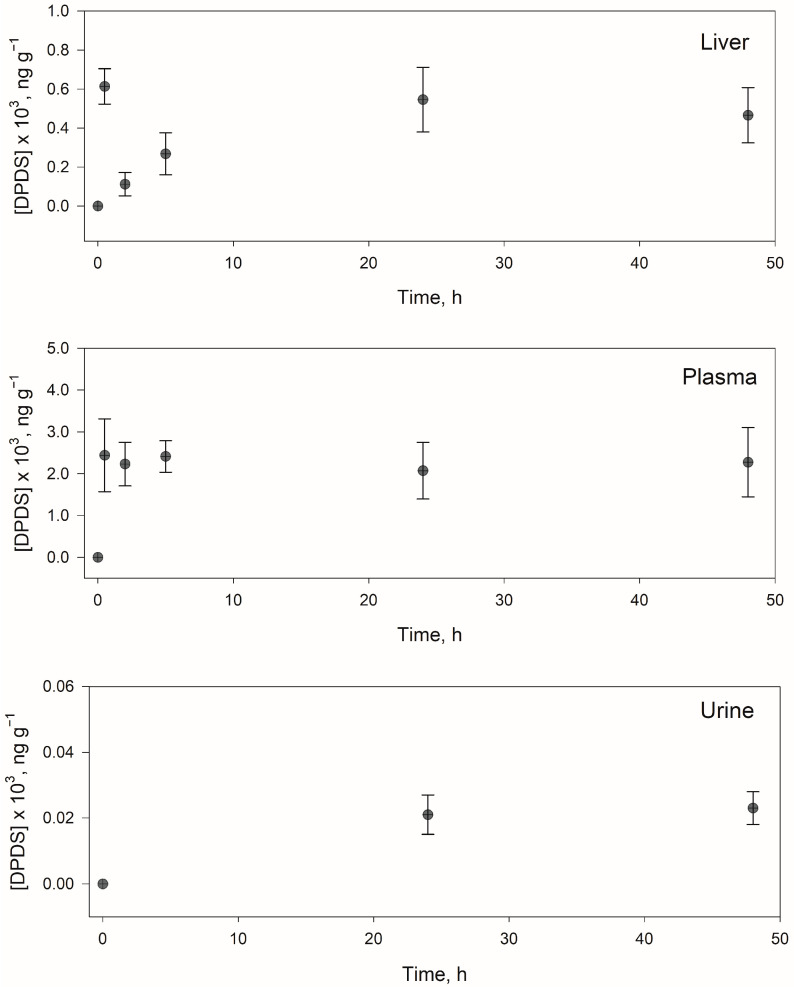
DPDS concentration found (ng g^−1^) in the different types of samples (liver, plasma, and urine) over time.

In urine, the lower concentrations were obtained for PTSO and DPDS, compared with the other matrices, which could indicate that the excretion of PTSO requires a time greater than 24 h. Figure 5 shows the identification and quantification of DPDS and PTSO in plasma, and Figure 6 shows the evolution with the time of DPDS in different samples. It should be noted that PM, MPS, MPSO, MPSO_2_, acetylated-CSSP, and acetylated-GSSP, for which standard analytical were not available, were not detected in any analysed sample.

## 4. Conclusions

A new analytical platform based on LLE with GC–MS and SALLE with UHPLC-QTOF-MS techniques has been developed and applied to the study of PTSO derivatives formed during its metabolisation pathway. Both methods have been validated, establishing matrix-matched calibration curves and LODs that were, in all cases, adequate for all the compounds of the study. After digestive absorption, PTSO concentrations were found in plasma samples after 5 h with a concentration of 180 ng mL^−1^ and DPDS concentrations between 2100–2400 ng mL^−1^ throughout the sampling time, meaning that PTSO was distributed through the blood in the form of DPDS but also appeared without chemical modification after 5 h. In contrast, we only found that it biotransformed into DPDS in the liver over the whole exposure time with concentrations between 110 and 610 ng g^−1^. Regarding excretion, both PTSO and DPDS were present in urine at 24 and 48 h; both were at very low concentration levels compared to the contents described in the other matrices. Conversely, no PTS, CSSP, or GSSP above the LODs were found. Furthermore, none of the monitored metabolites for which no standards were available was detected. Despite further experiments being convenient to elucidate the complete metabolic pathway for the PTSO and its derivatives, our present work shows that no PTSO is accumulated in the liver at any time, and only 5 h after the intake, trace amounts were detected in plasma.

## Figures and Tables

**Table 1:** Chromatographic and detection characteristics of the compounds monitored by GC–MS and UHPLC-Q-TOF-MS.

GC-MS					
Compound	t_R_ (min)	Quantification Ion, *m*/*z*		Qualifier Ions, *m*/*z*	
DPDS	5.27	150		43, 108	
PTSO	6.91	76		41, 43	
**UHPLC-Q-TOF-MS**					
**Compound**	**t_R_ (min)**	**[M+H]^+^** **Theoretical, *m*/*z***	**[M+H]^+^** **Experimental, *m*/*z***	**Error (ppm)**	**Products ions, *m*/*z***
CSSP	2.359	196.0460	196.0461	−0.27	179.0195, 106.9984
GSSP	2.551	382.1101	382.1111	−2.61	74.0028, 84.0412
PTS	2.989	167.0559	167.0562	−1.9	64.9490, 73.0078

**Table 2:** Analytical characteristics of LLE with GC–MS procedure.

Compound	Linearity			Sensitivity		Accuracy (n = 9)	
	Slope ^a^	R^2^	Linearity Range ^b^	LOD ^b^	LOQ ^b^	Level 1, RSD ^c^, %	Level 2 RSD ^c^, %
Liver							
DPDS	403 ± 6	0.99	50–5000	13	43	8.3	6.8
PTSO	247 ± 14	0.98	150–5000	38	125	10.1	9.3
Plasma							
DPDS	1575 ± 32	0.99	50–5000	11	36	9.6	8.7
PTSO	530 ± 28	0.98	200–5000	57	190	9.9	8.8
Urine							
DPDS	2835 ± 53	0.99	50–5000	6	20	7.5	6.9
PTSO	929 ± 54	0.98	100–5000	30	100	8.1	7.1

^a^ Liver reported in g ng^−1^; urine and plasma reported in mL ng^−1^. ^b^ Liver reported in ng g^−1^; urine and plasma reported in ng mL^−1^. ^c^ Level 1: Liver concentration 500 ng g^−1^. Urine and plasma concentration 500 ng mL^−1^. Level 2: Liver concentration 3000 ng g^−1^. Urine and plasma concentration 3000 ng mL^−1^.

**Table 3:** Analytical characteristics of SALLE with UHPLC-QTOF-MS procedure.

Compound	Linearity			Sensitivity		Accuracy (n = 9)	
	Slope ^a^	R^2^	Linearity Range ^b^	LOD ^b^	LOQ ^b^	Level 1, RSD ^c^, %	Level 2 RSD ^c^, %
**Liver**							
CSSP	69 ± 2.5	0.99	250–2000	71	240	8.8	6.8
GSSP	69 ± 1.9	0.99	150–2000	41	140	9.7	7.5
PTS	270 ± 19	0.98	100–2000	21	70	9.5	6.6
**Urine**							
PTS	1592 ± 16	0.99	50–1000	10	33	9.3	8.9
CSSP	86 ± 3.7	0.99	200–1000	55	185	11.5	10.6
GSSP	281 ± 3.8	0.99	50–1000	12	41	11.6	10.8
**Plasma**							
CSSP	128 ± 12	0.99	150–1000	37	124	11.1	10.8
GSSP	439 ± 13	0.98	50–1000	9	30	10.9	9.7
PTS	4006 ± 29	0.99	25–1000	6	20	8.5	7.9

^a^ Liver reported in g ng^−1^; urine and plasma reported in mL ng^−1^. ^b^ Liver reported in ng g^−1^; urine and plasma reported in ng mL^−1^. ^c^ Level 1: Liver concentration 500 ng g^−1^. Urine and plasma concentration 300 ng mL^−1^. Level 2: Liver concentration 1000 ng g^−1^. Urine and plasma concentration 800 ng mL^−1.^

**Table 4:** Possible PTSO-derived products monitored by UHPLC-Q-TOF-MS.

Compound	Molecular Formula	Molecular Ion	*m*/*z*
PM	C_3_H_8_S	[M+H]^+^	77.0419
MPS	C_4_H_10_S	[M+H]^+^	91.0576
MPSO	C_4_H_10_OS	[M+H]^+^	107.0525
MPSO_2_	C_4_H_10_O_2_S	[M+H]^+^	123.0474
Acetylated-CSSP	C_8_H_15_NO_3_S_2_	[M+H]^+^	238.0566
Acetylated-GSSP	C_15_H_25_N_3_O_7_S_2_	[M+H]^+^	424.1207

**Table 5:** Analysis of the samples.

Time, h	Content ^a^, ng g^−1^ or ng mL^−1^	
	DPDS	PTSO
Liver		
0	ND	ND
0.5	610 ± 90	ND
2	110 ± 60	ND
5	310 ± 100	ND
24	520 ± 210	ND
48	530 ± 110	ND
Plasma		
0	ND	ND
0.5	2400 ± 900	ND
2	2200 ± 500	ND
5	2400 ± 400	180 ± 50
24	2100 ± 700	170 ± 20
48	2300 ± 800	190 ± 50
Urine		
0	ND	ND
24	21 ± 6	50 ± 40
48	23 ± 5	43 ± 6

^a^ Mean value ± standard deviation (n = 10). ND: No detected.

## Data Availability

Data is contained within the article.

## References

[1] Putnik P., Gabrić D., Roohinejad S., Barba F.J., Granato D., Mallikarjunan K., Lorenzo J.M., Bursać Kovačević D. (2019). An overview of organosulfur compounds from *Allium* spp.: From processing and preservation to evaluation of their bioavailability, antimicrobial, and anti-inflammatory properties. Food Chem..

[2] Pastor-Belda M., Arroyo-Manzanares N., Yavir K., Abad P., Campillo N., Hernández-Córdoba M., Viñas P. (2020). A rapid dispersive liquid-liquid microextraction of antimicrobial onion organosulfur compounds in animal feed coupled to gas chromatography-mass spectrometry. Anal. Methods.

[3] Vezza T., Algieri F., Garrido-Mesa J., Utrilla M.P., Rodríguez-Cabezas M.E., Baños A., Guillamón E., García F., Rodríguez-Nogales A., Gálvez J. (2019). The immunomodulatory properties of propyl-propane thiosulfonate contribute to its intestinal anti-inflammatory effect in experimental colitis. Mol. Nutr. Food Res..

[4] Kim D.K., Lillehoj H.S., Lee S.H., Lillehoj E.P., Bravo D. (2013). Improved resistance to *Eimeria acervulina* infection in chickens due to dietary supplementation with garlic metabolites. Br. J. Nutr..

[5] Peinado M.J., Ruiz R., Echávarri A., Rubio L.A. (2012). Garlic derivative propyl propane thiosulfonate is effective against broiler enteropathogens in vivo. Poult. Sci..

[6] Vezza T., Garrido-Mesa J., Diez-Echave P., Hidalgo-García L., Ruiz-Malagón A.J., García F., Sánchez M., Toral M., Romero M., Duarte J. (2021). *Allium*-derived compound propyl propane thiosulfonate (PTSO) attenuates metabolic alterations in mice fed a high-fat diet through its anti-inflammatory and prebiotic properties. Nutrients.

[7] Ruiz R., García M.P., Lara A., Rubio L.A. (2010). Garlic derivatives (PTS and PTS-O) differently affect the ecology of swine faecal microbiota in vitro. Vet. Microbiol..

[8] Cascajosa Lira A., Prieto A.I., Baños A., Guillamón E., Moyano R., Jos A., Cameán A.M. (2020). Safety assessment of propyl-propane-thiosulfonate (PTSO): 90-days oral subchronic toxicity study in rats. Food Chem. Toxicol..

[9] Sorlozano-Puerto A., Albertuz-Crespo M., Lopez-Machado I., Ariza-Romero J.J., Baños-Arjona A., Exposito-Ruiz M., Gutierrez-Fernandez J. (2018). In vitro antibacterial activity of propyl-propane-thiosulfinate and propyl-propane-thiosulfonate derived from *Allium* spp. Against gram-negative and gram-positive multidrug-resistant bacteria isolated from human samples. Biomed. Res. Int..

[10] Sorlozano-Puerto A., Albertuz-Crespo M., Lopez-Machado I., Gil-Martinez L., Ariza-Romero J.J., Maroto-Tello A., Baños-Arjona A., Gutierrez-Fernandez J. (2021). Antibacterial and antifungal activity of propyl-propane-thiosulfinate and propyl-propane-thiosulfonate, two organosulfur compounds from *Allium* cepa: In vitro antimicrobial effect via the gas phase. Pharmaceuticals.

[11] Martínez-Fernández G., Abecia L., Martín-García A.I., Ramos-Morales E., Hervás G., Molina-Alcaide E., Yáñez-Ruiz D.R. (2013). In vitro—in vivo study on the effects of plant compounds on rumen fermentation, microbial abundances and methane emissions in goats. Animal.

[12] Rabelo-Ruiz M., Ariza-Romero J.J., Zurita-González M.J., Martín-Platero A.M., Baños A., Maqueda M., Valdivia E., Martínez-Bueno M., Peralta-Sánchez J.M. (2021). *Allium*-based phytobiotic enhances egg production in laying hens through microbial composition changes in ileum and cecum. Animals.

[13] Llana-Ruiz-Cabello M., Gutiérrez-Praena D., Puerto M., Pichardo S., Moreno F.J., Baños A., Nuñez C., Guillamón E., Cameán A.M. (2015). Acute toxicological studies of the main organosulfur compound derived from *Allium* sp. intended to be used in active food packaging. Food Chem. Toxicol..

[14] Mellado-García P., Puerto M., Prieto A.I., Pichardo S., Martín-Cameán A., Moyano R., Blanco A., Cameán A.M. (2016). Genotoxicity of a thiosulfonate compound derived from *Allium* sp. intended to be used in active food packaging: In vivo comet assay and micronucleus test. Mutat. Res.—Genet. Toxicol. Environ. Mutagen..

[15] Mellado-García P., Maisanaba S., Puerto M., Llana-Ruiz-Cabello M., Prieto A.I., Marcos R., Pichardo S., Cameán A.M. (2015). Genotoxicity assessment of propyl thiosulfinate oxide, an organosulfur compound from *Allium* extract, intended to food active packaging. Food Chem. Toxicol..

[16] Teyssier C., Siess M.-H. (2000). Metabolism of dipropyl disulfide by rat liver phase I and phase II enzymes and by isolated perfused rat liver. Drug Metab. Dispos..

[17] Germain E., Semon E., Siess M.H., Teyssier C. (2008). Disposition and metabolism of dipropyl disulphide in vivo in rat. Xenobiotica.

[18] Abad P., Lara F.J., Arroyo-manzanares N., Baños A., Guillamón E., García-campaña A.M. (2015). High-performance liquid chromatography method for the monitoring of the *Allium* derivative propyl propane thiosulfonate used as natural additive in animal feed. Food Anal. Methods.

[19] Abad P., Arroyo-manzanares N., García-campa A.M., Martinez-ferez A. (2018). Effects of different vehiculization strategies for the *Allium* derivative propyl propane thiosulfonate during dynamic simulation of the pig gastrointestinal tract. 2019, 253, 244–253. Can. J. Anim..

[20] Abad P., Arroyo-Manzanares N., García-Campaña A.M. (2016). A rapid and simple UHPLC-ESI-MS/MS method for the screening of propyl propane thiosulfonate, a new additive for animal feed. Anal. Methods.

[21] Mellado-García P., Puerto Rodríguez M., Prieto Ortega A.I., Pichardo S., Jiménez Morillo N.T., González-Pérez J.A. (2016). Determination of propyl propane thiosulfonate in plasma and tissues by UHPLC–MS/MS orbitrap and analytical pyrolysis (Py-GC-MS). Toxicol. Lett..

[22] Zhang G., Ll B., Lee C.H., Parkin K.L. (2010). Cysteine and glutathione mixed-disulfide conjugates of thiosulfinates: Chemical synthesis and biological activities. J. Agric. Food Chem..

[23] Sánchez C.J., Martínez-Miró S., Ariza J.J., Madrid J., Orengo J., Aguinaga M.A., Baños A., Hernández F. (2020). Effect of alliaceae extract supplementation on performance and intestinal microbiota of growing-finishing pig. Animals.

[24] Decision C. (2002). Commission Decision 2002/657/EC, Commission Decision 2002/657/EC of 12 August 2002 implementing Council Directive 96/23/EC concerning the performance of analytical methods and the interpretation of results. Off. J. Eur. Union.

